# The Voices of Schizophrenia as One’s Own Thoughts and What to Do About Them

**DOI:** 10.1093/schizbullopen/sgaf014

**Published:** 2025-08-25

**Authors:** Anna Cornelia Beyer

I am a scientist with schizophrenia since 2007. For a long time, I speculated that the voices of schizophrenia are telepathic. But recently, I have come to another conclusion. The voices of schizophrenia might be one’s own thoughts getting audible. For some reason, the brain might make one’s own thoughts appear as coming from an external source.

I speculate this because I started to realize recently that I can change what the voices say with a conscious effort of thought. So, if the voices say, for example: “I really hate you. God, how I hate you!,” then I consciously make an effort to think: “I love you, you are a good person, I am proud of you” etc. and the voices start to repeat these thoughts. They start to say: “I love you, you are a good person, I am proud of you” etc. Apparently, these voices are my own thoughts.

Apparently, it might be what is sometimes called our own inner critic that one hears with voices that comment on oneself especially. Oftentimes, these voices are very hard for patients to bear, because they are negative and not supportive, which might indicate a lack of self-love. This might mean, learning to consciously love oneself (and others, hating less), observing one’s thoughts constantly, which is both taught in meditation practices (loving-kindness meditation, for example), might be beneficial. One might be able to change the content of the voices with conscious effort of thought.

This approach does not always work, when the voices are very loud and intense on rare days I did not yet manage to apply it. But on many days it does work. And especially and additionally loving-kindness meditation leaves me blissful and peaceful. I have practiced this meditation for several months now, and with this reduced the voices to a considerable degree, and in addition made me happier and kinder. The voices are now quieter (so that they are not audible when music plays) and less frequent, and I even have days entirely without them. Due to loving-kindness meditation, days with voices that I cannot control become rarer and rarer, and my behavior, relationships, and well-being have improved significantly. I am told by friends that recently I made a lot of progress in my recovery, which I was not told before.

Loving-kindness meditation involves focusing benign thoughts toward both oneself and others and finally all humans or all living beings. It also involves activating the heart. In a deep state of relaxation, one focuses on quietly sending thoughts toward oneself and others such as: “May you be happy, may you be healthy, may you be loved.” There are many variations of this practice, and many guided loving-kindness meditations available on YouTube (and maybe other video platforms also). Loving-kindness meditation improves self-love and inspires more kindness toward others. It teaches to change negative, hateful, thoughts into positive, loving, thoughts. With this, it is similar to the approach mentioned at the start of this article.

